# Assigning cause for sudden unexpected infant death

**DOI:** 10.1007/s12024-014-9650-8

**Published:** 2015-01-30

**Authors:** Carl E. Hunt, Robert A. Darnall, Betty L. McEntire, Bruce A. Hyma

**Affiliations:** 1 Department of Pediatrics, Uniformed Services University of the Health Sciences, 4301 Jones Bridge Road, Bethesda, MD 20814-4799 USA; 2 Geisel School of Medicine at Dartmouth, Hanover, NH USA; 3 American SIDS Institute, Naples, FL USA; 4 Miami-Dade Medical Examiner Department, Miami, FL USA

**Keywords:** Sudden unexpected infant death, Sudden infant death syndrome, Infant mortality

## Abstract

We have reached a conundrum in assigning cause of death for sudden unexpected infant deaths. We summarize the discordant perspectives and approaches and how they have occurred, and recommend a pathway toward improved consistency. This lack of consistency affects pediatricians and other health care professionals, scientific investigators, medical examiners and coroners, law enforcement agencies, families, and support or advocacy groups. We recommend that an interdisciplinary international committee be organized to review current approaches for assigning cause of death, and to identify a consensus strategy for improving consistency. This effort will need to encompass intrinsic risk factors or infant vulnerability in addition to known environmental risk factors including unsafe sleep settings, and must be sufficiently flexible to accommodate a progressively expanding knowledge base.

## History of the term SIDS

Sudden infant death syndrome (SIDS) was originally defined in 1969 [1], focusing attention on sudden death in infants without an identified cause. These infants had a similar age at death and a strong association with sleep in common. Naming the sudden death SIDS instead of calling it “cause unknown” facilitated an enhanced focus on parental support and on research. Later studies identified prone sleep as a significant risk factor for SIDS-classified deaths [2–4]. The definition of SIDS was expanded in 1991, with an emphasis on scene investigation [5]. Although further modifications have been recommended [6–9], no consensus has been achieved. Indeed, a review of recent publications reported that the 1969 definition continues to be used 7 % of the time, the 1991 definition 35 % of the time, other modifications 26 % of the time and in 20 % no definition was mentioned [10].

Initially there were no candidate etiologies to explain these deaths. In the intervening years, however, much has been learned about environmental, biological, and genetic risk factors for deaths classified as SIDS. Once prone sleep was identified as a significant risk factor, most developed countries implemented back-to-sleep campaigns [11]. SIDS-classified death rates started to decline after 1990, as did non-SIDS-classified postneonatal mortality rates, and continued to decline until 2001 [11]. Much of this decline was initially attributed to an overall decrease in SIDS-classified deaths. Since 2001, however, SIDS-classified death rates have not continued to decrease whereas there has been a diagnostic shift to other assigned causes of sudden unexpected infant death or to unknown cause. Illustrating the impact of how these deaths are classified, SIDS-classified deaths declined by 20 % from 2005 to 2011, whereas for the same period the rate of accidental infant deaths increased by 5 % and rates for undetermined/unclassified deaths also increased [12, 13].

## The dilemma

With improvements in death scene investigations, including doll reenactment, medical examiners have been increasingly reluctant to assign SIDS (ICD-10 Code R95) as the cause of death. Adding to their reluctance was the epidemiologic identification of modifiable risk factors such as maternal cigarette smoking, bed-sharing, and soft bedding. One viewpoint is that since “SIDS” is by definition a diagnosis by exclusion, it should not be assigned as the cause of death if a risk factor consistent with possible asphyxia is present. In such instances, the cause assigned may be accidental suffocation or strangulation in bed (ASSB; ICD-10 Code W75) [11, 12]. Another viewpoint is that “SIDS” should not be assigned as the cause of death because every death must have a cause and if the postmortem investigation has not yielded one, it should be classified as “unknown” (ICD-10 Code R99). This is consistent with the approach to investigating and assigning cause of death in older children and adults. Some medical examiners object to the use of the term “syndrome” since there is no pattern of medical findings present at routine autopsy. In other cases, there may be a minor abnormality found at autopsy such as occasional foci of bronchopneumonia or limited residual findings related to bronchopulmonary dysplasia, and the death classified as having resulted from that finding. Finally, others are unwilling to assign the cause as SIDS unless all components of the definition have been satisfied, including an adequate death scene investigation.

Many deaths currently being classified as accidental suffocation are the same deaths classified as SIDS in past decades [14]. Studies of SIDS-classified deaths have identified environmental factors that are potentially asphyxiating as important risk factors, and there is thus an understandable overlap of many of the risk factors for deaths classified as SIDS and as ASSB [2, 3]. In reality, however, in many cases when such risk factors are present, there is no clear physical evidence of fatal airway compromise. Moreover, there are no objective criteria for fatal suffocation unless the scene investigation indicates obvious wedging or strangulation such that an underlying vulnerability would have been unnecessary to cause death. Otherwise, the conclusion that the death is caused by a lethal asphyxiating environment is based on circumstantial evidence of variable degrees of certainty. The key question is whether or when an unsafe sleep environment would be sufficient by itself to cause fatal asphyxia in the absence of an underlying vulnerability. Would most infants in the same environment have died?

More recently, the terms “SUID” and “SUDI” have been introduced to encompass all sudden *unexpected* infant deaths or sudden *unexpected* deaths in infancy, including those that are both explained and unexplained. Sudden unexpected deaths that remain *unexplained* after complete postmortem evaluation are considered by many to be equivalent to deaths previously classified as SIDS. Currently, there is no ICD-10 Code for “SUID” and any death with the descriptors “SUID” or “SUDI” is assigned the ICD-10 Code R95 for SIDS [13, 15]. The term “SUID” becomes further confused when some medical examiners use it to refer to infant deaths where no cause of death is found (i.e., *unexplained* instead of *unexpected*).

## Interactions between infant vulnerability and unsafe sleep environment

A common scientific explanation of “SIDS,” embodied in the triple risk model introduced by Filiano and Kinney in 1994 [16], is that it results from an interaction between infant vulnerability, a critical stage of development, and some exogenous “trigger” or stressor. Exogenous sleep-related stressors include prone position, over-bundling, bed sharing, and soft bedding, which are either singly, or in combination, *potentially* asphyxiating. The concept of a critical maturational or developmental period is derived from the peak incidence of these deaths in early infancy [2, 3]. The concept of vulnerability encompasses any intrinsic condition that might impair an infant’s ability to respond to significant environmental and/or positional asphyxia encountered during sleep.

**Fig. 1:**
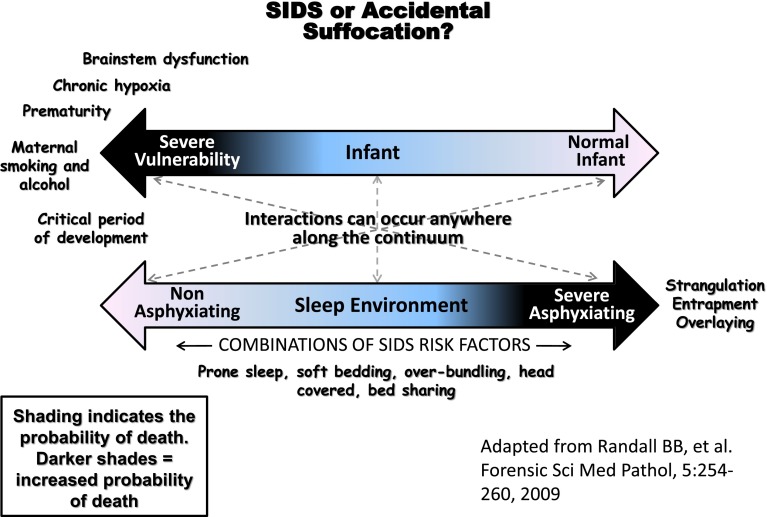
Schematic illustration of the spectrum of variability in risk for sudden unexpected infant death and the spectrum of variability in the degree of risk of the sleep-related environment, ranging from completely safe (non-asphyxiating) to potentially severe asphyxiating [42]

The importance of the relationship between infant vulnerability and environment is illustrated in the Fig. 1. In this model, there are interactions between two continua: (1) infant vulnerability and (2) a potentially asphyxiating sleep environment. Importantly, interactions can occur anywhere along the continua. Thus, a completely normal infant could die in a severely asphyxiating environment and an extremely vulnerable infant could die in a completely non-asphyxiating environment. Most deaths, however, occur between these two extremes.

A major advance in understanding the pathophysiology of SIDS-classified deaths was the discovery, using autoradiographic and immuno-histochemical methods, that a substantial subset of these infants has congenital, maturational, or acquired brainstem dysfunction that likely contributes to infant vulnerability [16, 17]. Importantly, these deficiencies were present in up to 70 % of SIDS-classified infants studied. These results have been confirmed in four independent data sets and by investigators in Australia and Japan [18, 19]. Kinney and colleagues have proposed that dysfunction in brainstem serotonergic and GABAergic mechanisms that control or modulate heart rate, breathing, body temperature, upper airway patency, sleep, and arousal, impair an infant’s ability to respond to stressors often encountered during sleep (Table 1) [17, 20–22]. These “protective” responses include arousal from sleep, cardiorespiratory responses to hypoxia and/or hypercapnia, motor responses required to lift and/or turn the head to clear the airway, the laryngeal chemoreflex, and autoresuscitation in response to asphyxia. Thus, infants with brainstem dysfunction would be expected to have an increased probability of death when faced with an adverse or unsafe sleep environment (See Fig. 1).

**Table 1:** Brainstem abnormalities reported in SIDS-classified deaths

Decreased muscarinic (acetylcholine) and kainate (glutamate) receptor binding in the arcuate nucleus
Decreased LSD (serotonergic receptor) binding in the caudal raphé, and other serotonergic regions
Decreased 5-HT_1A_ receptor binding, increased numbers of (especially immature) 5-HT neurons, a relative decrease in SERT binding
Decreased levels of 5-HT and TPH2, the major synthesizing enzyme for 5-HT
Decreased GABA_A_ receptor binding

*5*-*HT* 5-hydroxytryptamine (serotonin), *5*-*HT*_*1A*_ 5-HT receptor 1A, *SERT* serotonin transporter, *TPH2* tryptophan hydroxylase 2, *GABA*_*A*_ γ-aminobutyric acid receptor A

A number of other “intrinsic” risk factors for SIDS-classified deaths have also been identified that might affect brainstem and autonomic function, including fetal exposure to cigarette smoke, alcohol, cocaine, and other street drugs [2–4]. Prematurity also significantly increases risk for sudden unexpected death via unknown, but most likely maturational, mechanisms. For example, the combination of prematurity and bed-sharing has been found to substantially increase the risk for SIDS-classified deaths [23–25].

Francis Collins, Director of the NIH and past director of the Human Genome Research Institute noted in a presentation that “all illnesses have some hereditary contribution. Genetics loads the gun and environment pulls the trigger.” In SIDS-classified deaths, genes regulating physiological functions have been examined and summarized in recent reviews [25, 26] (Table 2). Polymorphisms have been related in particular to serotonin [26–31], cardiac channelopathies [32–34], and the autonomic nervous system [25, 26] but have also been identified in genes regulating inflammation and energy production [35–38]. Except for the channelopathies, the precise mechanisms by which these various polymorphisms might be a trigger for sudden infant death are unclear. Other limitations of the genetic studies include the limited power of some studies and lack of confirmation of others [39]. Indeed, even the reported serotonin gene variants may not have a significant role in the pathogenesis of SIDS-classified deaths, based on a lack of correlation with observed serotonin-related neuropathologic brainstem abnormalities [39].

**Table 2:** Categories of genes for which the distribution of polymorphisms differ in SIDS-classified deaths compared to controls

*Cardiac channelopathy polymorphisms*
Potassium ion channel genes *(KCNE2*, *KCNH2*, *KCNQ1)*
Sodium ion channel gene (*SCN5A*) (long QT syndrome 3, Brugada syndrome)
*GPD1*-*L* (Brugada syndrome)
SCN3B (Brugada syndrome)
*CAV3* (long QT syndrome 9)
SCN4B (long QT syndrome 10)
SNTA-1 (long QT syndrome 11)
*RyR2* (catecholaminergic polymorphic ventricular tachycardia)
*Serotonin polymorphisms (5*-*HT)*
5-HT transporter protein *(5*-*HTT)*
Intron 2 of *SLC6A4* [variable number tandem repeat (VNTR) polymorphism]
5-HT FEV gene
*Autonomic nervous system polymorphisms*
Paired-like homeobox 2a (PHOX2A)
PHOX2B
Rearranged during transfection factor (RET)
Endothelin converting enzyme-1 (ECE1)
T cell leukemia homeobox (TLX3)
Engrailed-1 (EN1)
Tyrosine hydroxylase (THO1)
Monamine oxidase A (MAOA)
Sodium/proton exchanger 3 (NHE3) (medullary respiratory control)
*Infection and inflammation polymorphisms*
Complement C4A
Complement C4B
Interleukin-1RN [gene encoding IL-1 receptor antagonist (ra); pro-inflammatory]
Interleukin-6 (IL-6) (pro-inflammatory)
Interleukin-8 (pro-inflammatory; associated with prone sleeping position)
Interleukin-10 (IL-10)
Vascular endothelial growth factor (VEGF) (pro-inflammatory)
Tumor necrosis factor (TNF)-α (pro-inflammatory)
*Other categories of polymorphisms*
Mitochondrial DNA (mtDNA) polymorphisms (energy production
Flavin-monooxygenase 3 (FMO3) (metabolizes nicotine; risk factor in mothers who smoke)

Except for the channelopathies, the mechanisms by which these polymorphisms lead to sudden death are not known. Many studies are of limited power, and not all have been confirmed

## Summary and call to action

There is strong evidence confirming unsafe sleep environment as a major risk factor for sleep-related sudden unexpected infant deaths. Thus we need to continue to expand and enhance our public health education efforts, including more effectively overcoming persisting cultural, historical, and other behavioral and socio-demographic barriers to safe sleep for all infants. We must strengthen our research efforts to identify underlying pathophysiology and predictive markers that might further prevent these tragic deaths. Unfortunately, however, the inconsistent approach to assigning cause of death will persist until consensus can be achieved among the various relevant disciplines.

The reasons for the lack of broad-based acceptance of any new approach to classification [7–9] are unclear and likely multifactorial. At a minimum, however, contributions to this lack of consensus include the diversity in international medical and legal approaches to assigning cause of death, and failure to include all of relevant professional and public disciplines in the discussion.

We recommend that an interdisciplinary international committee be organized to review current approaches for assigning final cause of death, and to identify a consensus strategy for improving consistency. This effort may best be coordinated by an international organization or individuals without a vested interest in a particular outcome but having the requisite credibility and consensus-building ability. Since medical examiners and coroners have the difficult task of classifying infant deaths, they need to have a prominent role in this consensus process, as do the government agencies responsible for tabulating vital statistics. Without a fully participatory interdisciplinary international process, we will not achieve the broad-based comprehensive endorsement required for successful acceptance and implementation.

In summary, we need a more consistent approach to assigning cause of death that is not only consistent with our current understanding of environmental risks contributing to an unsafe sleeping environment but also with interactions with maturational and biologic vulnerability including genetic risk factors. Rapidly evolving advances in genetic technologies including next-generation sequencing and other systems biologic approaches including metabolomics should lead to progressive expansion of our knowledge of relevant genetic and gene–environment interactions and the identification of predictive markers [40, 41]. We thus need an approach to classification that not only addresses current inconsistencies but that is sufficiently flexible to accommodate new knowledge enhancing our understanding of the complex interactions resulting in sleep-related sudden unexpected infant death.
